# Investigating exceedances of formaldehyde levels and source identification in offices of an academic medical institute

**DOI:** 10.1093/joccuh/uiae049

**Published:** 2024-08-14

**Authors:** Watcharakorn Chuthong, Vithawat Surawattanasakul, Ratana Sapbamrer, Wachiranun Sirikul

**Affiliations:** Department of Preventive and Social Medicine, Faculty of Medicine, Chulalongkorn University, Bangkok, Thailand; Department of Community Medicine, Faculty of Medicine, Chiang Mai University, Chiang Mai, Thailand; Environmental and Occupational Medicine Excellence Center, Faculty of Medicine, Chiang Mai University, Chiang Mai, Thailand; Department of Community Medicine, Faculty of Medicine, Chiang Mai University, Chiang Mai, Thailand; Environmental and Occupational Medicine Excellence Center, Faculty of Medicine, Chiang Mai University, Chiang Mai, Thailand; Department of Community Medicine, Faculty of Medicine, Chiang Mai University, Chiang Mai, Thailand; Environmental and Occupational Medicine Excellence Center, Faculty of Medicine, Chiang Mai University, Chiang Mai, Thailand

**Keywords:** formaldehyde, indoor air pollution, office worker

## Abstract

Objectives: To investigate factors associated with indoor formaldehyde levels in office settings within an academic medical institute.

Methods: This cross-sectional study was conducted in 25 offices (261 workers) at a medical university in Thailand. Questionnaires gathered data on demographics, work patterns, and office equipment usage (printers, photocopiers, air fresheners, liquid paper, glue, cleaning agents, and marker pens). The building environment was assessed by a multidisciplinary team. Formaldehyde levels and relevant parameters (temperature and relative humidity) were measured in each room both indoors and outdoors. A multiple linear regression model investigated the relationship between formaldehyde and office factors, controlling for room conditions.

Results: Median office formaldehyde levels were 442.1 μg/m^3^ (interquartile range: 343.8-908.7 μg/m^3^), exceeding World Health Organization and Thai guidelines. Photocopier use was significantly associated with higher levels of indoor formaldehyde (β = .20; 95% CI, 0.30-0.37; *P* = .02). Air freshener use also showed a significant association (β = .56; 95% CI, 0.30-0.81; *P* < .001). No correlation was found between the use of liquid paper, glue, printers, cleaning agents, or marker pens and indoor formaldehyde levels.

Conclusions: Indoor formaldehyde levels in these offices exceeded the established guidelines. Use of photocopiers and air fresheners was associated with increased formaldehyde levels. Implementing interventions such as improved ventilation and regular screening is essential for creating healthier office environments.

## Teaser

Key points: **• What is already known on this topic:** Formaldehyde is an indoor air pollutant commonly found in building materials, furnishings, and various household products. However, knowledge about specific sources of formaldehyde in office environments, particularly in academic medical settings, remains limited.

• What this study adds: This study shows high formaldehyde levels in academic medical offices. Photocopiers and air fresheners are identified as potential sources.

• How this study might affect research, practice, or policy: Routine indoor formaldehyde monitoring and targeted mitigation strategies are crucial for protecting office worker health and well-being.

## 1. Introduction

In recent decades, the significance of indoor air quality has gained prominence, given that a substantial 80%-90% of daily activities occur indoors.^1^ Although global attention has focused on pollutants such as indoor particulates (PM_2.5_ and PM_10_), ozone, NO_2_, SO_2_, and CO since the World Health Organization (WHO) guidelines were published in 2005, leading to stricter regulations in many countries, there are persistent challenges.^2^ Despite improvements in these areas, indoor air still harbors various chemical substances that pose health risks. Among these, volatile organic compounds (VOCs), notably formaldehyde, remain a cause for concern due to their established health implications.^3^

Formaldehyde, a colorless and pungent gas, displays remarkable chemical reactivity at room temperature and readily forms formic acid when reacting with hydroxyl radicals.^3^ Formaldehyde is associated with a range of health effects, predominantly irritating the skin, eyes, and respiratory tracts.^3^ In 2005, the INDEX project^4^ reported concentrations causing mild eye irritation and perceived odor starting from 30 μg/m^3^. The International Agency for Research on Cancer classified formaldehyde as a Group 1 human carcinogen in 2004, specifically due to its link to nasopharyngeal cancer and leukemia.^5^ Formaldehyde can be found ubiquitously in both outdoor and indoor environments. Outdoor origins include combustion processes, vehicle emissions, and industrial activities. Meanwhile, indoor formaldehyde emanates from diverse sources such as wood-based products, tobacco smoke, insulation materials, and indoor chemistry, all of which have the potential to release this compound.^6^ Exposure to formaldehyde is significantly higher indoors compared with outdoors, primarily attributed to stronger emission sources and lower air exchange rates in indoor environments.^7^ Several studies have examined formaldehyde concentrations in indoor settings, providing valuable insights.

For instance, Raw et al^8^ conducted a study of formaldehyde levels in residential bedrooms, reporting a geometric mean of 22.2 μg/m^3^ and a maximum value of 171 μg/m^3^. In the AIRMEX project, formaldehyde concentrations inside buildings and kindergartens in European cities were found to be 7-8 times higher than outdoor levels, ranging from 3 to 35 μg/m^3^ in public buildings and offices.^9^ Similarly, a Taiwanese study based on continuous monitoring, revealed that mean formaldehyde concentrations in offices ranged from 75 μg/m^3^ to 300 μg/m^3^, with occasional short-term peaks reaching up to 1000 μg/m^3^. The primary cause of high formaldehyde concentrations in offices was identified as office materials.^10^ Among various indoor environments, the office setting emerges as a prominent microenvironment where individuals spend most of their working hours.

Several studies have explored potential sources of various indoor air pollutants emitted by office equipment, including ozone, VOCs, and particulate matter.^6^^,^^11^ Of particular concern is formaldehyde, as several investigations have revealed that electronic devices such as printers and photocopiers can be significant sources of formaldehyde emissions in indoor air.^12^^,^^13^ Additionally, consumer products like cleaning agents and air fresheners can elevate indoor formaldehyde levels, both through direct emissions and as secondary organic aerosols.^14^^-^^16^

In our previous study, we discovered a high presence of formaldehyde in the offices of a medical university where air conditioning was in use and there was no smoking. This presence was associated with sick building syndrome among the office workers.^17^ Although the adverse effects of formaldehyde on the health of office workers are well documented, identifying the specific sources of formaldehyde within office environments remains a challenging task. This study aimed to evaluate the factors that are correlated with indoor formaldehyde levels in office settings.

## 2. Materials and methods

### 2.1. Population and setting

**Figure 1:**
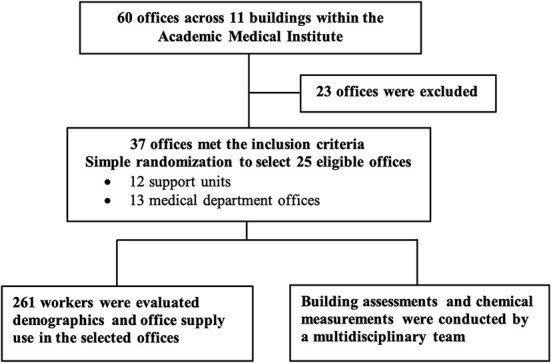
The study flow diagram.

In October 2021, a cross-sectional study was performed at an academic medical institute to investigate the relationship between indoor formaldehyde levels and potential sources in an office environment. The facility management unit of the institution provided information regarding the office workers and offices. To be eligible for inclusion, administrative offices with at least 3 workers and air conditioning were selected, whereas 23 offices were excluded due to their specific tasks. In this study, a simple random sampling technique was employed to select a sample of 25 offices from a total of 37 eligible offices across multiple departments. The selected offices included 12 out of 15 support units and 13 out of 22 medical departments. Data were collected from all office workers in these selected offices (Figure 1).

### 2.2. Questionnaire and target potential factors

A self-administered questionnaire was used to collect data on the usage of relevant potential sources in the office environment. The questionnaire was distributed to participants within 2 weeks following assessments of the working environment and the use of office supplies. A total of 261 workers (90%) completed the questionnaire. The questionnaire consisted of 3 sections. The first section collected general participant information, including gender (male/female), age (years), and smoking status (never smoker, former smoker, current smoker). The second section focused on work information, collecting data on department and division, office location (including building name, floor number, and room number), employment duration (years), and regular work hours and overtime (hours per day and days per week). Finally, the third section addressed potential sources of formaldehyde by asking participants about their use of various office supplies in the past 3 months. These supplies included liquid paper, glue, photocopiers, printers, air fresheners, cleaning agents, and marker pens. The data collection form was developed by the research team and is presented in Supplementary material 1.

### 2.3. Building environment assessment

A multidisciplinary team conducted the office environment investigation. The team comprised occupational medicine physicians, an occupational health nurse, an industrial hygienist, a safety officer, an engineer, and an environmental toxicologist. An assessment form was employed to gather observational data on various aspects of the office environment within each room. The form consisted of 3 sections including building characteristics, room characteristics, and air quality measurements. The building characteristics section collected information such as building name, age, and number of floors. The room characteristics section documented details including floor number, room number, department, division, room dimensions (width and length), number of occupants, stagnant air areas, and air conditioning usage and system details. Finally, the air quality measurement section documented both indoor and outdoor air quality data, including temperature, relative humidity, and formaldehyde levels. The detailed assessment form can be accessed in Supplementary material 2.

### 2.4. Air quality measurement

This study focused on assessing indoor formaldehyde levels and related parameters using established techniques and tools validated in our prior research.^17^ Key details of the measurement procedures are summarized as follows. Air temperature and relative humidity were measured with a Q-TRAK Indoor Air Quality Monitor. Formaldehyde concentrations were assessed using a MIRAN SapphIRe portable interface ambient air analyzer. All instruments were calibrated according to manufacturers’ specifications to ensure measurement accuracy and reliability. Our indoor air quality index (IAQ) measurements followed the Singapore Standard Council for IAQ 2021 guidelines.^18^ Assessment points were positioned at a height of 75-120 cm above the floor, on a clear table with a 30-minute sampling duration. The number of assessment points was determined based on room size, with 1 point designated per 500 m^2^, positioned in the middle of the designated area. No personnel were allowed in the vicinity until the collection process was completed. Outdoor air quality measurements were collected for reference at a single location and time, typically at the center of all selected buildings for 30 minutes, to establish a baseline for comparison. Recorded air quality measurement data were meticulously logged.

### 2.5. Statistical analysis

To analyze the general information, work information, potential sources of formaldehyde, building and room characteristics, and air quality measurement a descriptive analysis was conducted. Categorical variables were presented using frequency and percentage, whereas continuous variables were presented as appropriate. A median with an interquartile range (IQR) and a mean with an SD were used to describe parametric data and nonparametric data, respectively. Multiple linear regression analysis was employed to investigate the association between indoor formaldehyde levels and potential factors within the office, adjusting for room conditions. The adjusted variables included room area per occupant (square meters per person), temperature (°C), relative humidity (%), and area with no air movement. The results of the analysis were expressed as adjusted b values, accompanied by a 95% CI and a significance level of *P* < .05. The variance inflation factor (VIF) was calculated to confirm that the analysis was not affected by multicollinearity. The findings of the study were reported in accordance with the STROBE (Strengthening the Reporting of Observational studies in Epidemiology) guidelines.^19^

### 2.6. Ethical consideration

This study was approved by the Institutional Review Broad at the Faculty of Medicine, Chiang Mai University (ID: 8477/2021; date of approval: September 15, 2021).

## 3. Results

### 3.1. General participant information and work information

A total of 261 participants were included in the study. Females comprised the majority of participants (59.0%). The average participant age was 40 years, and most participants were nonsmokers (86.2%). The average employment duration among participants was 9 years. Regular working hours averaged 37 hours per week, with a median overtime of 2 hours per week. Details are presented in Table 1.

**Table 1:** General participant and work information.

**Characteristics (*n* = 261 participants)**	
**General participant information**	
**Gender, *n* (%)**	
**Male**	107 (41.0)
**Female**	154 (59.0)
**Age, y, mean ± SD**	40 ± 11
**Smoking, *n* (%)** **Nonsmoker** **Ex-smoker** **Current smoker**	225 (86.2) 24 (9.2) 12 (4.6)
**Work information**	
**Employment duration, y, median (IQR)**	9 (2-20)
**Regular working hours per week, mean ± SD**	37 ± 8
**Overtime working hours per week, median (IQR)**	2 (0-5)

Abbreviation: IQR, interquartile range.

### 3.2. Building characteristics, room characteristics, and indoor air quality parameters

As seen in Table 2, the average building age was 35 years (IQR, 18-49 years) with an average of 9 floors (IQR, 7-9 floors). Among the 25 eligible office rooms, the median room area per occupant was 8.2 m^2^ per person (IQR, 5.8-10.6 m^2^). Only 2 rooms (8%) lacked proper air circulation. All offices turned off air conditioning after office hours, with only 1 room turning it off during working hours. The majority of offices maintained a regular service schedule for air conditioning maintenance and cleaning. The indoor air quality measurements showed a median temperature of 23.3°C and a relative humidity of 62.2%. The median indoor formaldehyde concentration was 442.1 μg/m^3^. The recorded concentrations ranged from a minimum of 0 μg/m^3^ to a maximum of 3096 μg/m^3^. During the study period, the outdoor meteorological conditions included an average air temperature of 30.2°C and a relative humidity range of 54.7% to 67.3%. The average outdoor formaldehyde concentration was 0.04 μg/m^3^.

**Table 2:** Building and room characteristics and indoor air quality parameters.

**Characteristics (*n* = 25 rooms)**	
**Building characteristics**	
**Age of the building, y, median (IQR)**	35 (18-49)
**Number of floors in the building, median (IQR)**	9 (7-9)
**Room characteristics**	
**Number of occupants per room, median (IQR)**	19 (10-28)
**Room area per occupant, m** ^**2**^ **, median (IQR)**	8.2 (5.8-10.6)
**Areas with no air movement, *n* (%)**	2 (8.0)
**Air conditioning for room, *n* (%)**	
**Turned off during office hours**	1 (4.0)
**Turned off after office hours**	25 (100.0)
**Air conditioning system service for room, *n* (%)**	
**Scheduled maintenance**	23 (92.0)
**Maintained**	23 (92.0)
**Cleaned**	23 (92.0)
**Indoor air quality parameters**	
**Indoor air temperature, °C, median (IQR)**	23.3 (22.9-24.1)
**Indoor relative humidity, %, median (IQR)**	62.2 (55.8-66.4)
**Indoor formaldehyde level, μg/m** ^**3**^, **median (IQR)**	442.1 (343.8-908.7)
**Number of rooms formaldehyde level exceeds the standard limit, *n* (%)**	20 (80)
**Number of rooms formaldehyde level does not exceed the standard limit, *n* (%)**	5 (20)

### 3.3. Factors correlated with indoor formaldehyde levels by multiple linear regression analysis

Table 3 provides further information about the levels of use of potential sources of formaldehyde in office rooms. Printers were the most frequently used office equipment (87.0%), whereas air fresheners were used the least (9.6%). Multiple linear regression was performed to discover the correlated factors in offices with indoor formaldehyde levels, including room characteristics, indoor air indices, and potential sources in office rooms. Of the 261 participants, the results showed a significant positive correlation between indoor formaldehyde levels and air fresheners, as indicated by a significant b coefficient of .56 (95% CI, 0.30-0.81). Furthermore, photocopiers also significantly increased indoor formaldehyde levels with a b coefficient of .20 (95% CI, 0.03-0.37). There were no significant increases in indoor formaldehyde levels associated with using liquid paper, glues, printers, cleaning agents, or marker pens. There was no evidence of multicollinearity, as each variable had a VIF below 2, with an average mean VIF of 1.2.

**Table 3:** Multiple linear regression analysis of factors associated with indoor formaldehyde levels (*n* = 261 participants).^a^

**Potential source**	**Users, *n* (%)**	**b**	**95% CI**		***P* value** ^**b**^
			**Lower bound**	**Upper bound**	
Liquid paper	153 (58.6)	−0.02	−0.18	0.14	.77
Glue	103 (39.5)	−0.07	−0.23	0.09	.41
Photocopier	164 (62.8)	**0.20**	**0.03**	**0.37**	.02^*^
Printer	227 (87.0)	0.00	−0.22	0.22	.97
Air freshener	25 (9.6)	**0.56**	**0.30**	**0.81**	<.001^**^
Cleaning agent	54 (20.7)	0.12	−0.05	0.30	.17
Marker pen	125 (47.9)	0.07	−0.05	0.23	.21
Constant		0.15	−0.06	0.37	.15

a Adjusted variables: room area per occupants (square meters per person), temperature (°C), relative humidity (%), area with no air movement.

b ^*^Significant association at *P* value .05; ^**^significant association at *P* value .001. Bolded rows indicate sources that are statistically significantly correlated with indoor formaldehyde levels.

## 4. Discussion

In this study, we observed that the office rooms exhibited a median formaldehyde concentration of 442.1 μg/m^3^ (IQR, 343.8-908.7 μg/m^3^). Formaldehyde is a prevalent indoor and outdoor air pollutant,^9^ commonly found indoors in buildings within the range 7-79 μg/m^3^. However, the concentrations of formaldehyde in our office settings exceeded the WHO’s recommended guideline,^3^ which advocates a 30-minute average concentration below 100 μg/m^3^. The elevated formaldehyde levels found in our study are of significant concern, particularly for office workers who spend a considerable amount of time in indoor environments. Such heightened exposure to formaldehyde can lead to sensory irritation and may have long-term health implications, including an increased risk of cancer.^3^ This level also surpasses the acceptable threshold recommended by the Thai authorities, which limits formaldehyde exposure to 120 μg/m^3^ in indoor environments.^20^ Furthermore, our previous research indicated a significant correlation between formaldehyde exposure at this level and the prevalence of sick building syndrome among the population studied.^17^

**Figure 2:**
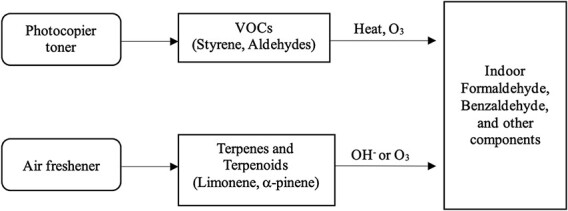
Possible indoor-related formaldehyde sources.

In our research, we observed higher indoor formaldehyde concentrations compared with various international studies examining formaldehyde levels in office environments. For instance, in a large-scale European study conducted by the European Indoor Air Monitoring and Exposure Assessment Project (AIRMEX) between 2003 and 2008, the average formaldehyde levels in offices were reported as 16.9 μg/m^3^ in Ireland, 9.0 μg/m^3^ in the Netherlands, 17.9 μg/m^3^ in Greece, 11 μg/m^3^ in Finland, 24.3 μg/m^3^ in Germany, and 16.2 μg/m^3^ in Hungary.^9^^,^^21^ Meanwhile, the US Environmental Protection Agency’s Building Assessment Survey and Evaluation (BASE) study reported an average formaldehyde level of 16.9 μg/m^3^ in office spaces.^21^ However, a study in Taiwan^10^ reported short-term peaks of formaldehyde reaching up to 1000 μg/m^3^. In Thailand,^22^ a separate study found the mean formaldehyde concentration in offices to be 35.5 μg/m^3^. The primary indoor sources of formaldehyde in offices are attributed to cleaning products, printers, air fresheners, and various office equipment, which either directly emit formaldehyde or contribute to its generation as secondary pollutants.^6^^,^^11^ Other potential sources, such as smoking, can also elevate indoor formaldehyde levels due to the reaction of styrene, acrolein, and nicotine from tobacco with environmental ozone.^23^^-^^25^ However, smoking was excluded from the assessment because it is prohibited in Thai universities. There was no formalin used in any of the rooms selected for this study. Furthermore, none of these rooms were located near anatomy classrooms, pathology laboratories, or forensic laboratories where formalin is used. Additionally, there was no wallpaper installation or carpeting in these offices, and no recent structural renovations were performed in these rooms. Therefore, these sources can be assumed not to interfere with formaldehyde levels in this study. Outdoor formaldehyde plays a minimal role in influencing indoor formaldehyde levels in this study due to the air conditioning systems used in the buildings: split-type and central air systems. Neither system exchanges indoor air with outside air. However, air exchange between indoor and outdoor environments is still possible when workers leave windows or doors open.

Photocopiers have emerged as a significant source of formaldehyde emissions in this study, with a b coefficient of .20 (95% CI, 0.30-0.37; *P* = .02). Numerous studies have explored formaldehyde levels during photocopier operation, consistently revealing a positive correlation.^26^^-^^28^ Two primary factors have been identified as contributors to formaldehyde emissions: ozone and VOCs. The latter are present in certain toners, particularly those containing solvents such as styrene and aldehydes, and play a crucial role in the formaldehyde emission process. When photocopiers are in use, these VOCs are released as the toner is heated, leading to the generation of formaldehyde and other by-products.^12^^,^^26^^,^^29^ Even when photocopiers are in idle mode, a low VOC emission persists due to their presence in toners.^29^ Ozone, another significant factor, is produced from the copying process.^27^^,^^28^^,^^30^ When photocopiers operate, ozone can react with styrene and aldehydes, resulting in the formation of formaldehyde and benzaldehyde as by-products.^23^ Outdoor ozone infiltration might also contribute to this reaction, although photocopiers themselves do not emit ozone when idle.^26^^,^^31^ The extent of ozone emissions from photocopiers depends on various factors, including room volume, ventilation, temperature, usage time, and the surfaces in the vicinity. These variables collectively influence the release of ozone during the operation of photocopiers.^11^^,^^30^^,^^32^^,^^33^ Studies have shown that formaldehyde levels from photocopiers can sometimes be undetectable or remain at low concentrations.^32^ The variation in emitted compounds can be attributed to the use of different toner formulations in dry-process photocopy machines.^27^

Terpenoids, such as limonene and α-pinene, act as the primary precursors in reactions with ozone, leading to the indoor generation of formaldehyde by air fresheners.^14^^,^^23^^,^^34^^-^^36^ These terpenoids are commonly found in oils or scent formulations used in various products, including α-terpinene from pine oil and selected sesquiterpenes present in waxes, orange oil, and lemon-peel oil.^14^ Numerous studies have revealed a connection between air freshener use and increased formaldehyde levels due to ozone interaction with terpenes, resulting in stable products like formaldehyde, acetone, ultrafine particles, and other secondary organic compounds.^15^^,^^34^^-^^36^ Formaldehyde can also be a product derived from reactions between unsaturated organic compounds and hydroxyl radicals.^15^ The formation of formaldehyde and acetone, but not acetaldehyde, is consistent with the occurrence of unsaturated carbon–carbon bonds in the reactive VOC constituents of these products.^14^^,^^16^ Emissions of primary constituents depend on factors like product composition, concentration, and usage patterns. Ventilation also plays a crucial role in determining indoor pollutant concentrations. Notably, a gap exists in research comparing emissions from inkjet and laser printers. Human factors, including frequency, amount, and manner of application, also influence emissions.^14^ Although formaldehyde concentrations may cause sensory irritation, the reviewed studies do not consistently demonstrate well-characterized respiratory symptoms related to exposure.^34^^,^^37^^,^^38^ Nevertheless, our study found a significant association between air fresheners and formaldehyde levels, with a b coefficient of .56 (95% CI, 0.30-0.81; *P* < .001). Although air fresheners emit terpenoids at lower rates, resulting in substantially lower concentrations compared with cleaning products containing these compounds, it is essential to consider the continuous emission characteristic of plug-in air fresheners. This continuous release may lead to more chronic exposures to secondary pollutants over time.^15^ The process of formaldehyde release is described in Figure 2.

Printer toner is widely recognized as a source of various aldehyde emissions, including formaldehyde, and the presence of aldehydes like benzaldehyde has also been identified as due to oxidation products.^13^^,^^23^ The mechanism of formaldehyde production in printers is similar to that in photocopying, involving the interaction between toner and ozone.^11^^,^^39^ Despite this, variations in concentration and emission rates are expected due to background effects.^32^ However, our study found no significant correlation between office printers and indoor formaldehyde levels (b coefficient = 0; 95% CI, −0.22 to 0.22; *P* = .96). This outcome is likely due to the diversity of toner types used, which can contribute to varying emission products. Cleaning products share similar precursors, such as limonene and other terpenoids, which react with ozone similarly to air fresheners.^23^ Formaldehyde emissions can also originate from additives like formaldehyde-releasing preservatives and disinfectants, which release aldehydes like formaldehyde and glutaraldehyde.^14^^,^^36^^,^^40^ In this study, we did not find a significant association between formaldehyde levels and cleaning products; the b coefficient was 0.12 (95% CI, −0.05 to 0.30; *P* = .017). The disparities in ingredients, concentrations, and usage patterns could account for these differing outcomes compared with other studies. Regarding the formaldehyde-emission mechanism from liquid paper, glue, and marker pens, the specific pathways remain unclear. Our study did not establish any association between formaldehyde levels and these items. Notably, most glue used for stationery is formaldehyde-free, with urea-formaldehyde glue predominantly used in wood adhesives. These differences in glue formulations may explain the lack of significant formaldehyde emissions from glues used in office settings. Recommendations aimed at reducing indoor formaldehyde levels in offices should align with the hierarchy of controls concept. These recommendations include the significant use of formaldehyde-free or formaldehyde precursor-free substances in photocopier toners and air fresheners. Implementing engineering controls, such as enhancing ventilation systems, is advisable. Additionally, administrative controls, such as relocating photocopier machines to unoccupied rooms or using air fresheners before or after working hours, can be considered.

We identified some limitations in this study. Firstly, the cross-sectional design limited our ability to establish causality between different parameters. Temporal ambiguity prevented the establishment of causal relationships. Secondly, the lack of documentation on construction materials prevented us from determining their exact constituents. Thirdly, the absence of documentation on the production year of the furniture in the buildings surveyed prevented us from identifying whether furniture was old or new, which both could be potential sources of elevated indoor formaldehyde levels.^6^ These omissions limit the comprehensiveness of our findings. Fourthly, certain local and human activity-related determinants, such as ingredient composition, concentration, and patterns of usage, were not identified or considered in the study design. Expanding the scope to encompass a wider range of potential sources could enhance the accuracy of our results. Despite these limitations, our study underscores that formaldehyde remains a significant concern in office environments that warrants resolution.

## 5. Conclusions

This study identified photocopiers and air fresheners as significant sources of indoor formaldehyde emissions in office environments. Proper ventilation remains a crucial engineering solution to mitigate these risks. Additionally, future interventions should focus on reducing formaldehyde emissions from these sources at the product level. Real-time data collection for pre- and post-intervention comparisons will be essential to assess the effectiveness of these interventions in lowering formaldehyde exposure and its associated health risks. Implementing effective preventive measures is crucial to ensure a healthier indoor environment and safeguard the well-being of occupants in office settings and similar enclosed spaces. Further research should explore additional potential sources of formaldehyde emissions, such as off-gassing from new furniture materials.

## Supplementary Material

Web_Material_uiae049

## Data Availability

The data underlying this article will be shared on reasonable request to the corresponding author.
