# Supplementary material for: Investigating exceedances of formaldehyde levels and source identification in offices of an academic medical institute
Source: J Occup Health. 2024 Aug 14;66(1):uiae049. doi: 10.1093/joccuh/uiae049 (PMC11457052; doi:10.1093/joccuh/uiae049)
Supplement: Web_Material_uiae049 [file web_material_uiae049.zip › 240810_revision_Office_Environment_Assessment_and_Chemical_Measurement_Form.docx]

**Office Environment Assessment and Air Quality Measurement Form**

Form Number ___________

Date __________________

**1.Building Characteristics**

Building Name _________________

Age of building ____________________ Number of floors ____________________

**2.Room Characteristics**

Floor ___________ Room Number _____________

Department _______________________ Division _______________________________

Room width (m) _____________________ Room length (m) _________________________

Number of occupants ____________________

Are there any areas in the room with no air movement? Yes No

Is the air conditioning typically turned off during office hours? Yes No

Is the air conditioning typically turned off after office hours? Yes No

Air Conditioning System Service:

Air Conditioning System Maintenance Schedule Yes No

Air Conditioning System Maintenance (Actual) Yes No

Air Conditioning System Cleaning Schedule Yes No

**3.Air Quality Measurement**

|  | Room | Outdoor* |
| --- | --- | --- |
| Temperature (°C) |  |  |
| Relative humidity (%) |  |  |
| Formaldehyde level(μg/m^3^) |  |  |

*Outdoor chemical measurements used for reference are typically collected at a single location and time
