# Supplementary material for: Investigating exceedances of formaldehyde levels and source identification in offices of an academic medical institute
Source: J Occup Health. 2024 Aug 14;66(1):uiae049. doi: 10.1093/joccuh/uiae049 (PMC11457052; doi:10.1093/joccuh/uiae049)
Supplement: Web_Material_uiae049 [file web_material_uiae049.zip › [JOH] 240710 revision Office Supply Usage Questionnaire.docx]

Questionnaire Number _____

**Office Supply Usage Questionnaire** Date ____ / _____ / ____

**General Participant Information**

1. Gender Female Male

2. Age _______

3. Smoking: No Smoking but quit

Current smoker

**Work Information**

1. Department ___________________ Division __________________

2. Office Building Name ________________ Floor __________ Room Number _____________

3. Employment duration (year) _______________________

4. **During regular work hours**, on average, I typically occupy this office room _______ hours per day and _______ days per week.

5. **Outside of regular work hours**, on average, I typically occupy this office room _______ hours per day and _______ days per week.

**Potential sources of formaldehyde**

1. In the **past 3 months**, have you used the following items while working in the office?

1.1. Liquid paper Yes No

1.2. Glue Yes No

1.3. Photocopier Yes No

1.4. Printer Yes No

1.5. Air freshener Yes No

1.6. Cleaning agent Yes No

1.7. Marker pen Yes No
